# The role of Bergmann glial cells in cerebellar development

**DOI:** 10.1186/2049-3002-1-13

**Published:** 2013-06-11

**Authors:** Pierre Leprince

**Affiliations:** 1 GIGA-Neuroscience, University of Liège, CHU B36, Liège B4000, Belgium

## Correspondence

A response to Gershon et al. : **Hexokinase-2-mediated aerobic glycolysis is integral to cerebellar neurogenesis and pathogenesis of meduloblastoma**. *Cancer and Metabolism* 2013, **1**(2):17

Gershon et al. [1] have investigated the role that Hexokinase-2 (Hk2) plays in the control of aerobic glycolysis during cerebellar neurogenesis and pathogenesis of medulloblastoma. They conclude from experiments using cerebellar granule neurons progenitors (CGNPs) in culture, dissected normal cerebella and medulloblastoma and cerebellar slices that Hk2 mediates a glycolytic phenotype in proliferating CGNP that is recapitulated in medulloblastoma, a malignant tumor of CGNP origin. While the role of Hk2 in the aggressive growth of medulloblastoma is well documented in their work, its importance during the proliferation, migration and differentiation of cerebellar granule neurons may have been overestimated by not taking into account the metabolic activity and histogenetic role of Bergmann glial cells, a major actor of cerebellar development. Indeed the Bergmann glia palisade is already fully developed and occupies most of the cerebellar cortex between pia and the Purkinje cells layer at P7 in the mouse at a time when Gershon et al. claim that CGNPs are mostly responsible for an elevated Hk2 activity in the cerebellum. In their figure 3, this area where Hk2 is expressed the most at P7 that they have labeled as EGL (external granule layer), actually corresponds to the Molecular Layer (ML) in which Bergmann glia send their dense, branched processes. The EGL sensu stricto is made up of a 4 to 8 cells-thick layer located at the superficial part of the ML, and CGNPs proliferate very actively only in the outermost half of that EGL. This is visible in figure 6H of the paper of Gershon et al., where the proliferating CGNPs are stained in green, while the post-mitotic CGNPs are stained in red. Beyond that, CGNPs cell bodies are only sparsely present in the ML as they rapidly migrate toward the internal granule layer (IGL) along Bergmann glia processes.

Gershon et al. interpretation of the results of their Fig. 5 may also be discussed as the hGFAP-cre;Hk2^fl/fl^ conditional knockout that they have used to study the metabolism and migratory behavior of CGNPs during their development not only targets those neurons [2], but will also affect gene expression in Bergmann glia since these cells are among those in post-natal mice in which the hGFAP promoter is expressed most [3]. Thus any conclusion that is inferred from this model about the effect of Hk2 knockdown on the glycolytic metabolism and migratory behavior of CGNPs, may also be applied to Bergmann glia. In particular, since Bergmann glia processes guide the migration of post-mitotic CGN past the Purkinje cell layer to the EGL and constitute the cerebellar glia limitans that cover the EGL and conceivably helps to organize it, any alteration of the energy metabolism of Bergmann glia resulting from Hk2 knockdown is likely to impact strongly on the way that the associated CGN behave during their migration.

One might thus feel that in their work, Gershon et al. may have overlooked an important aspect of the cerebellar developmental process and one could suggest that they give more attention to the possible involvement of Bergmann glia in modulating the role of Hexokinase-2-mediated aerobic glycolysis during cerebellar neurogenesis.
